# Influence of Nonuniform Exciton Density on Diffusion Length Measurements via Photoluminescence Quenching

**DOI:** 10.1021/acsomega.5c05659

**Published:** 2025-11-26

**Authors:** Bruno Guilherme Araujo Pimenta, Tiago de Sousa Araújo Cassiano, Ricardo Gargano, Pedro Henrique de Oliveira Neto

**Affiliations:** † Institute of Physics, 664468 University of Brasília, 70919-970 Brasília, Brazil; ‡ International Center of Physics, 28127 University of Brasília, 70919-970 Brasília, Brazil

## Abstract

Addressing the challenge of energy efficiency in organic photovoltaics (OPVs) requires investigating exciton transport mechanisms. Exciton migration in OPVs is influenced by factors such as the morphology, temperature, absorption properties, and excitation conditions. In this context, the diffusion length (*L*_D_) is a key parameter that characterizes overall transport efficiency. Spectroscopic techniques, particularly photoluminescence quenching, are commonly used for *L*_D_ measurements. However, in bilayer quencher setups, these measurements can be limited by assumptions about exciton behavior, potentially leading to inaccurate *L*_D_ estimates. In this work, we assess the magnitude of error in *L*_D_ measurements resulting from incorrect exciton generation assumptions. It was found that the error largely depends on the molecules’ absorption characteristics. An analysis of common organic compounds suggests errors of up to 30%. The findings reveal an important limitation in commonly adopted experimental protocols that estimate the diffusion length. Moreover, assessing the error magnitude might aid in the interpretation of future experimental characterizations, furthering the understanding of exciton dynamics in practical setups.

## Graphical abstract

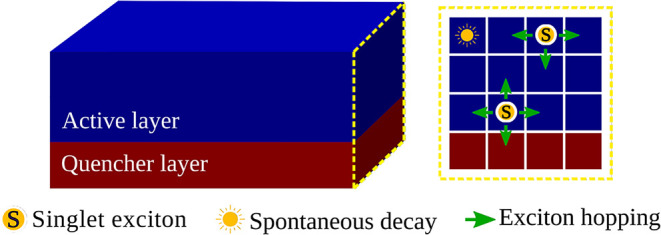

## 1 Introduction

Organic photovoltaics (OPVs) setups are one of the most promising candidates for renewable energy sources, owing to their significant low cost, lightweight, and flexibility.^1–3^ Significant progress toward industrial use has been made through important advances in stability^4,5^ and efficiency.^6^ Today, operational OPVs with efficiencies nearing 20% are a reality,^6–8^ offering an encouraging prospect for future optoelectronics.

The OPV operation involves the diffusion of bound electron–hole pairs, known as excitons.^9^ Therefore, from an efficiency perspective, the focus revolves around understanding exciton migration.^10,11^ Toward this task, accurate experimental and theoretical characterizations are essential. Specifically, the diffusion length (*L*_D_) is commonly used as a metric to measure a key aspect of the OPV’s potential, playing a central role in validating and directing new OPV architectures.

Fundamentally, exciton diffusion consists of dynamical competition among transfer, recombination, and emission processes. The förster resonance energy transfer (FRET) is the leading theory that models intermolecular exciton hopping,^12^ relating the transfer rate to the molecule’s optoelectronic properties. One way to connect this description with the diffusion mechanism is by numerically simulating the transport through the kinetic Monte Carlo (KMC) algorithm.^13,14^ In this formalism, hopping and decay events are represented as rates given by FRET theory.^15,16^ Through this protocol, morphological effects^11,17^ as well as complex recombination processes such as singlet–triplet conversions,^18^ thermally activated delayed fluorescence,^19,20^ and annihilation^14,21–25^ can be taken into account dynamically. Importantly, the approach allows for direct calculation of the diffusion length.^21,26^

Experimentally, *L*_D_ can be estimated through the attenuation of photoluminescence (PL) when mixing the material with quenchers.^12,27–29^ In bilayer quenchers, the film thickness modulates the attenuation. Effectively, this shifts the quencher interface farther from the illuminated side of the film, thereby adjusting the available diffusion path. To link the PL results with *L*_D_, one common approach is to fit the analytic solution of the diffusion equation to recover the experimental quenched PL spectrum.^27,28,30–32^

However, the standard analytic solution^28,30,32,33^ is formally valid only when exciton generation is homogeneous. In other words, the exciton density does not have a spatial dependence immediately after photon absorption. While some setups can be engineered to approximately reach this state, this does not necessarily apply to general configurations.

Recently, Belova et al.^34^ investigated the influence of geometrical and optical effects on exciton *L*_D_ measurements using the surface PL quenching method. They examined how different multilayer architectures affect the extracted *L*_D_ values. Markedly distinct optical behaviors were observed across these structures, revealing a strong geometry-dependent modulation of the light outcoupling. As a result, the reliability of such methods has become a matter of concern. Notably, a simple change in the refractive index of the quencher layer was shown to cause a 3-fold increase in the extracted *L*_D_. This underscores a critical limitation in established protocols, which may fail to account for extrinsic optical effects that mask intrinsic exciton dynamics.

The conditions of exciton generation are a similar matter. In general, indirect measurements that rely on fitting procedures are inherently dependent on the acquired data. This makes them vulnerable to artificial agreement, particularly when the data set is limited. Consequently, determining the validity of assuming uniform generation during experiments becomes challenging. Ultimately, it remains unclear how significantly the experimentally obtained *L*_D_ values may deviate when this assumption is not met.

The purpose of this work is to provide a tangible notion of the error introduced to diffusion length estimates when uniform exciton creation is assumed. To this end, we simulate the exciton dynamics in bilayered quencher films for a number of generation regimes, progressively relaxing the homogeneous condition. The simulations cover a wide range of parameters, representing the organic class. Results show that the absorption profile might heavily influence the error in *L*_D_, leading to variations of up to 30%. Additionally, we report the presence of a significant error sensitivity depending on the excitation pulse wavelength. In other words, the choice of the irradiation wavelength can improve (or diminish) the *L*_D_ measurement precision. This work provides insight into diffusion length experiments, aiding the interpretation of results and suggesting routes to minimize the error.

## 2 Methodology

### A Diffusion Equation

The standard dynamical model for exciton transport consists of the following one-dimensional diffusion equation for the exciton density *n*(*z*, *t*)^27,30,35^

1 $$\frac{\partial n\left(z,t\right)}{\partial t}=D\frac{\partial^{2}n\left(z,t\right)}{\partial z^{2}}-\frac{n\left(z,t\right)}{\tau }$$

where *D* is the diffusivity and τ is the average decay time. Knowing these two parameters for a particular system enables the calculation of the diffusion length

2 $$L_{D}=\sqrt{\tau D}$$

### B Analytical FRET-Based *L*_D_ Estimate

According to the Förster theory, the rate at which an exciton is transferred from a donor molecule **D** to a neighboring acceptor **A** is^12,17,19,25,26^

3 $$k_{F}=\frac{1}{\tau_{\mathrm{emi}}}{\left(\frac{R_{F}}{r_{0}}\right)}^{6}$$

where τ_emi_ is the exciton radiative lifetime and *r*_0_ is the intermolecular lattice parameter. The parameter *R*_F_ is known as the Förster radius and is defined as

4 $$R_{F}^{6}=\frac{9c^{4}\kappa^{2}\tau_{\mathrm{emi}}}{8\pi }\int_{0}^{\infty }\frac{I_{D}\left(\omega \right)\sigma_{A}\left(\omega \right)}{\omega^{4}}d\omega$$

in which ω is the photon frequency, σ_**A**_(ω) is the acceptor cross section, *I*_**D**_(ω) is the donor differential emission rate, *c* is the speed of light, and κ^2^ is the orientation factor, which corresponds to 2/3 for amorphous crystals.^36^ Complementarily

5 $$k_{\mathrm{emi}}=\frac{1}{\tau_{\mathrm{emi}}}=\int_{0}^{\infty }I_{D}\left(\omega \right)d\omega$$

is the fluorescence rate.

For an isotropic transport where periodic boundary conditions (PBC) are in place, the diffusion length expressed in the eq 2 can be written in terms of FRET theory (*L*_D_^*F*^)^26^

6 $$L_{D}^{F}=\sqrt{D\tau_{\mathrm{em}i}}=\sigma \left(|r|\right)=\overset{®}{\left|r\right|}=\frac{R_{F}^{3}}{r_{0}^{2}}$$

where σ­(|*r*|) and $\overset{®}{\left|r\right|}$ are, respectively, the exciton standard deviation and the average absolute displacement. Moreover, under this regime, it can be shown that the exciton displacement from its initial position follows a mean-free Laplace distribution

7 $$f\left(z;\sigma \right)=\frac{1}{2\sigma }\exp\left(-\left|\frac{z}{\sigma }\right|\right)$$

This distribution can serve as a reference to identify how different a particular diffusion regime is from isotropic transport. In our model, the distributions along the *x̂* and *ŷ* directions reduce to eq 7, since periodic boundary conditions are imposed in those directions.

In real systems, nonradiative losses are always present. Treated as a monomolecular process, they share the same mathematical form as fluorescence eq 5

8 $$k_{\mathrm{nrad}}=\frac{1}{\tau_{\mathrm{nrad}}}$$

where *k*_nrad_ and τ_nrad_ denote the nonradiative decay rate and lifetime, respectively. Since both processes follow the same form, an effective decay time can be defined as

9 $$\tau_{\mathrm{total}}={\left(1/\tau_{\mathrm{emi}}+1/\tau_{\mathrm{nrad}}\right)}^{-1}=\frac{\tau_{\mathrm{emi}}\tau_{\mathrm{nrad}}}{\tau_{\mathrm{emi}}+\tau_{\mathrm{nrad}}}$$

which represents the average exciton lifetime in a pristine material before decay. The only consequence is a predictable reduction in the diffusion length *L*_D_^*F*^,^37^ while the overall exciton dynamics remain unchanged. Accordingly, nonradiative events are not explicitly addressed in this work, as they can be readily incorporated when interpreting the diffusion results, not requiring explicit simulation.

### C Quencher-Based *L*_D_ Estimate

The simulated idealized experimental setup is represented in Figure 1. Two materials are arranged in a bilayer configuration. The first one, in blue, symbolizes the material of interest, while the quencher is the second material (red), intersecting at a distance of *L*. The typical exciton dynamics begin after irradiation of the device. The molecules that absorb the photons become excited, giving rise to excitons. Then, the excitations start to migrate throughout the blue material. Those that reach the interface between the two materials can hop into the quencher zone. If the transfer occurs, then the excitation does not contribute to the final PL spectra, resulting in attenuated fluorescence. For this configuration, the fluorescence intensity *I*(*L*; *L*_D_) is given by

10 $$I\left(L;L_{D}\right)=\int_{0}^{\infty }\int_{0}^{L}n\left(z,t\right)dzdt$$

where contributions from the axes parallel to the interface have already been considered.

**Figure 1:**
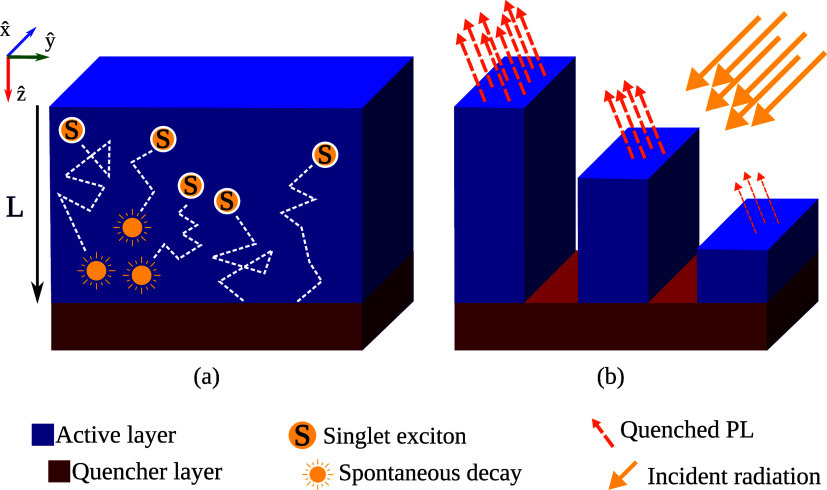
Schematic representation of the experimental setup for photoluminescence quenching in bilayer systems, showing the reference axes. (a) Dynamics of excitons in the active layer, including their decay via fluorescence or quenching at the quencher layer. (b) Modulation of the film photoluminescence as a function of the quencher position *L*.

Therefore, the quencher efficiency can be defined as the following ratio

11 $$Q\left(L;L_{D}\right)=1-\frac{I\left(L;L_{D}\right)}{I_{0}}$$

where *I*_0_ is the fluorescence intensity in the absence of a quencher. It can be shown from the solution of eq 1 under homogeneous generation^27,28,30,32,33^ that

12 $$Q\left(L;L_{D}\right)=\frac{L_{D}}{L}\tanh\left(\frac{L}{L_{D}}\right)$$

where *L*_D_ can be obtained by fitting the curve above using the PL spectra. A formal proof of this result can be found in the Supporting File.

In the experimental setup, the diffusion length can be calculated from spectroscopy measurements of bilayered quenching configurations. To represent this approach, we simulate the exciton dynamics by means of the kinetic Monte Carlo algorithm, thereby obtaining the quencher efficiency curve, as would be measured experimentally.

The KMC is a protocol to dynamically accept potential events that an exciton can perform using a stochastic approach.^21^ The probability of each event occurring directly depends on its rate. Under a low-density regime, bimolecular effects such as annihilation can be neglected. Consequently, it can be assumed that the carrier can either hop to a neighboring site or decay.^26,38–40^ The hopping and fluorescence rates are given by, respectively, eqs 3 and 5.

As for the morphology, the quenched bilayer configuration shown in Figure 1(a) is represented in the KMC simulation by defining two types of sites. The first, shown in blue, corresponds to the organic compound of interest, in which one aims to measure the *L*_D_. Inside this zone, the exciton can freely hop or decay. The other type of site is the quencher, representing the material in the red region shown in Figure 1(a). The quenching layer introduces a new event, which traps any exciton that eventually reaches this layer, forbidding fluorescence events and further transference. In practice, this corresponds to the removal of the exciton from the simulation. Consequently, they do not contribute to the PL spectrum. Additional details regarding the calculation of the quencher efficiency from the KMC output and the algorithm, in general, are present in the Supporting File.

Since we are interested in determining the effect of nonuniform generation on *L*_D_ estimates, the absorption mechanism from the irradiation source has to be considered. Here, we represent this effect through a semiclassical approach in which the exciton creation is modeled via an exponential function along the (*z*) direction that intersects the red and blue regions. The initial exciton density is modeled as^35^

13 $$n\left(z,t=0\right)=\rho_{0}e^{-\alpha z}$$

Here, ρ_0_ is the concentration at the first layer, and α is the inverse of the characteristic penetration length, the absorption coefficient.

It is important to realize that since bimolecular effects are neglected, ρ_0_ is merely a scaling factor in the fluorescence counts. Consequently, the following results should hold for an arbitrary ρ_0_, provided that the final number of simulated excitons is enough to mitigate statistical fluctuations in the diffusion results.

The initial condition is implemented in the KMC workflow by sampling the exciton population according to eq 13, which explicitly depends on the site position along the intersecting line. The closer α is to 0, the more uniform the creation of excitons becomes. In other words, the characteristic penetration length increases. Alternatively, a higher α corresponds to a higher population in the first layers at the initial instant. A sample of these initial generation curves for decreasing absorption coefficients is shown in Figure 2(a). In the limiting case of α = 0.0 *r*_0_^–1^, the homogeneous generation scenario is recovered, as all layers receive the same average number of excitons (purple curve in the inset), while no excitons are generated in the quencher region, with its interface located at *z* = 70 *r*_0_^–1^. Figure 2(b) shows the time evolution of one such curve for a fixed nonzero α, at times corresponding to fractions of the fluorescence decay time: 0.0, 0.2, and 1.0τ. After a full decay time, most excitons either have undergone fluorescence or been quenched.

**Figure 2:**
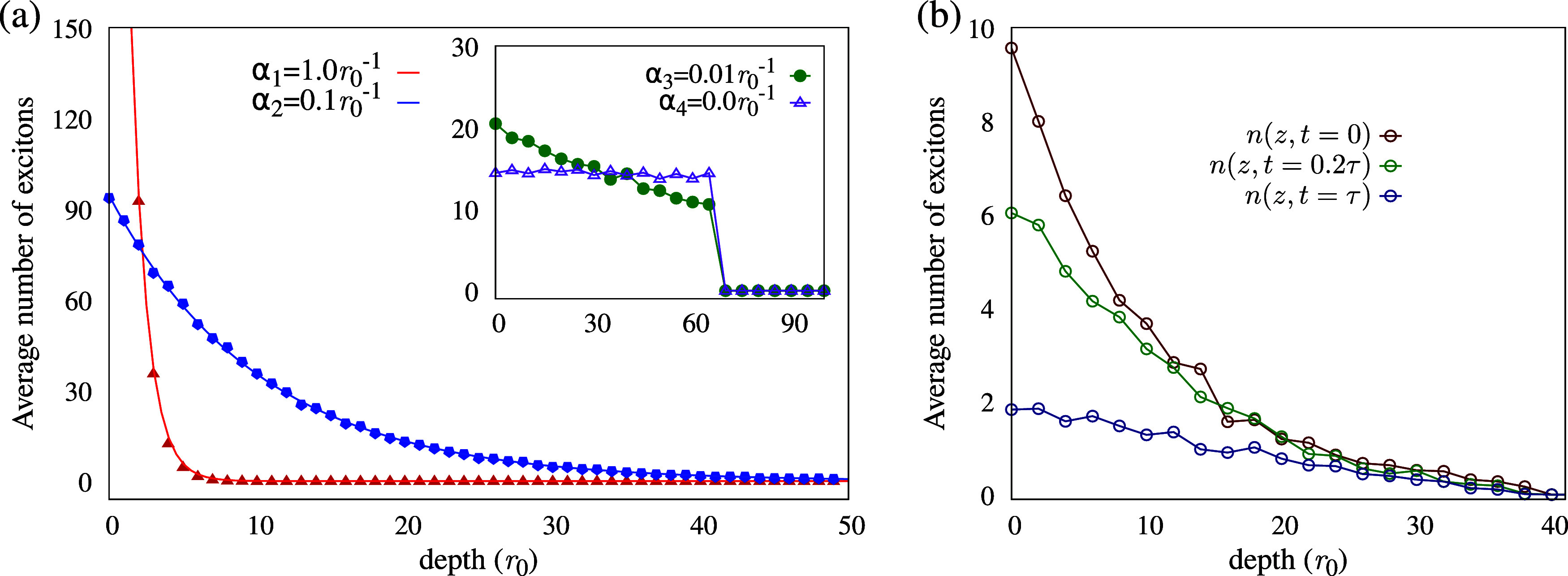
Visualization of exciton generation curves along the lattice’s *ẑ* direction. (a) Initial (*t* = 0) curves for various absorption coefficients are shown. The inset highlights the limiting case where α ≈ 0.0 *r*_0_^–1^, for which the decay becomes negligible. (b) Time evolution of a curve with fixed absorption is displayed.

Simulating the exciton dynamics via KMC gives access to the theoretically quenched PL spectra, allowing replication of the experimental protocol. Fitting the corresponding quencher efficiency curve returns a diffusion length estimate. Then, its precision can be put to the test by comparing it with the analytical results for any combination of *L* and α. Further details regarding the KMC implementation are provided in the Supporting File.

### D *L*_D_ Error Estimate

To assess the agreement between the quencher-based estimates and the reference values, we define the following percentage error

14 $$\Delta L_{D}\left(\alpha ;L_{D}^{F}\right)=\left|\frac{L_{D}^{F}-L_{D}}{L_{D}^{F}}\right|\times 100\%$$

in which we explicitly distinguish the FRET-based *L*_D_^*F*^ reference from the quencher-based fitted *L*_D_.

## 3 Results and Discussion

Our study begins by analyzing the effect of a quencher layer on the displacement distribution. Initially, we adopt a parametric approach to obtain a general estimate of the *L*_D_ error. The physical variables do not correspond to a specific molecule. Instead, we will consider a group of configurations. This set will collectively represent the organic material class. For this approach, it is convenient to parametrize the variables in terms of a single parameter. Here, we conveniently define all distance-related parameters in units of lattice parameter *r*_0_.

Another point is that the decay time in a purely diffusive setup does not affect the diffusion length. The reason is that the FRET transfer and fluorescence rates depend on τ_emi_ through the same term, 1/τ_emi_. Consequently, the number of fluorescence events per hopping event does not depend on τ_emi_. Therefore, a single value of τ_emi_ can be set and yields the same diffusion results. In fact, the FRET-based diffusion length in eq 6 does not depend explicitly on τ_emi_. Here, we set τ_emi_ = 1 ns for the sake of convenience. More generally, within the scope of our parametric investigation, any other value of τ_emi_ is effectively captured through a corresponding choice of *R*_F_, as incorporated in eq 4. Naturally, a more complex setup involving dynamical quasiparticle recombination or an active layer containing a mix of different materials would lift this symmetry, requiring a more elaborate choice of τ_emi_ values.

Before considering the effect on *L*_D_ of incorrectly assuming the generation hypothesis, it is important to investigate the effect of quenchers on the diffusion distributions. To this end, we present in Figure 3 a series of distributions in which quenchers and nonuniform generation are progressively introduced. In what follows, *dz* is the difference between the exciton’s initial *z* position and its position where fluorescence or quenching takes place.

**Figure 3:**
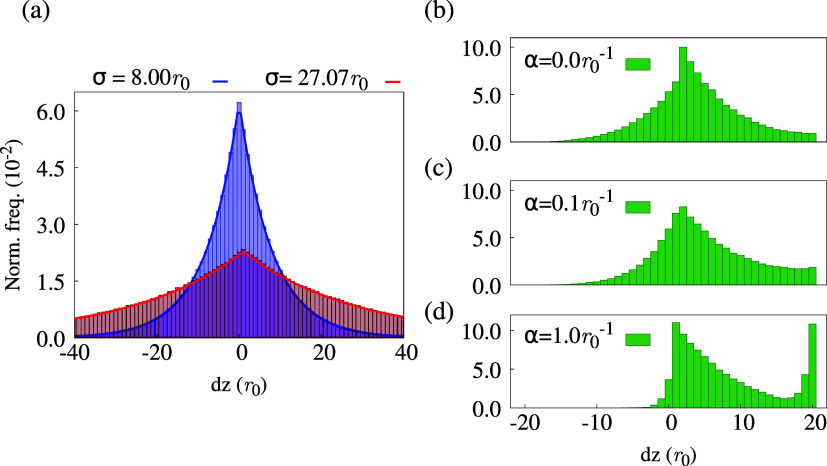
Normalized exciton displacement distributions in units of lattice parameter *r*_0_ without quencher, fitted with a Laplace distribution (a), and with the quencher at *L* = 20*r*_0_ (b–d). In part (a), we exhibit a low diffusivity case (blue, with *R*_F_ = 2.0*r*_0_ and *r* = 1.0*r*_0_) and a high diffusivity case (red, with *R*_F_ = 3.0*r*_0_ and *r* = 1.0*r*_0_), both within periodic boundaries. In parts (b–d), we display the distributions for the low diffusivity case and varying α.

First, we consider the reference configuration in which the creation is homogeneous, PBCs are active, and there is no quencher. Figure 3(a) shows two *dz* displacement distributions in this regime. The curve in blue corresponds to the configuration *R*_F_ = 2*r*_0_, while the red one refers to *R*_F_ = 3*r*_0_. As can be seen, the two curves exhibit the expected form of a mean-free Laplace distribution, as shown in eq 7.

Naturally, the red curve, which has a higher Förster radius, exhibits a wider dispersion than the blue curve, indicating a stronger diffusion. Another important confirmation is that the variance-calculated *L*_D_ values are in good agreement with the analytical FRET-based definition shown in eq 6. For instance, the red curve has a calculated *L*_D_ = 27.07*r*_0_, while the analytical result gives *L*_D_^*F*^ = 27*r*_0_. A similar comparison is possible with the other curve, where *L*_D_ = 8*r*_0_ and *L*_D_^*F*^ = 8*r*_0_. Recovering these fundamental physical traits through the simulations corroborates the methodology, validating the theoretical framework of the foregoing results.

Next, we consider the effect of adding a quencher layer while keeping the generation homogeneous, as shown in Figure 3(b). Here, *L* = 20*r*_0_ and *R*_F_ = 2.0*r*_0_. As can be seen, the quencher presence degrades the distribution’s reflection symmetry. Put another way, when *dz* = 0 is used as a reference, the left side is not a mirrored reflection of the right side. Negative *dz* values are related to diffusion away from the quencher, while the positive shift indicates motion toward it. Although the distribution decays smoothly for the *dz* < 0, the same is not true for the opposing side. Inside the *dz* > 0 region, the distribution abruptly drops to 0 after *dz* = 20*r*_0_, which is exactly the quencher position. That is a clear manifestation of the quencher’s presence, forbidding further displacement beyond a distance *L*. Therefore, adding the quencher in a homogeneous creation breaks the symmetry of the distribution, cutting off the positive side. In practice, this would result in a reduction of *L*_D_, which is expected, considering that the quencher can be seen as a physical barrier.

Enforcing nonhomogeneous creation enhances the distribution asymmetry. Figure 3(c,d) presents the case with α = 0.1*r*_0_^–1^ and α = *r*_0_^–1^, respectively. First, by our model, *n*(*z* = *L*, *t*) = 0, meaning that all distributions are cut off at this layer position. On a second analysis, the positive portion of the distribution gradually bulges, as shown in Figure 3(c), until it reaches an inversion point, as displayed in Figure 3(d). This is a direct consequence of the increase in α, which creates excitons farther from the quencher.

The observed transition can be intuited by analyzing the progression in the distribution illustrated in Figure 3(b,c). Two major changes appear: (1) the exciton counts for *dz* < 0 are attenuated; (2) there is a peak rising at *dz* = *L*.

The first effect is due to the proximity of an average exciton to the first layer of the material. No transport is allowed beyond the first layer. Consequently, excitons generated initially near this region cannot develop a motion with a large negative *dz*. Instead, they are limited to the distance between the first layer and the initial position. As α grows, this separation becomes narrower. For a sufficiently large α, most excitons begin at the first layer, making the displacement distribution contain negligible counts for *dz* < 0.

The reason for the second effect lies in the motion toward the quencher. For high values of α, all excitons begin nearly at the same *z* position, close to the first layer. If the Förster radius is comparable to (or higher than) *L*, a significant portion of the excitons is diffusive enough to reach the quencher. Because no motion is allowed beyond this region, two excitons that could cover different distances beyond the *dz* = *L* mark will be counted with the same *dz* = *L*, leading to the peak in this region.

Naturally, this effect is also fueled by the first layer’s constraint. Since the distribution is normalized by the number of excitations, the decay observed for *dz* < 0 must be compensated for elsewhere. Excitons that would travel large *dz* < 0 distances in a uniform creation are forbidden from going beyond the first layer. However, locally, their dynamics are essentially the same because the rates are identical regardless of the direction. Consequently, they will contribute to the distribution’s positive side.

Having established the direct effects of exciton generation and quencher layers on the diffusion mechanism, we can now consider how these conditions affect the *L*_D_ estimate using the quencher-based fitting approach. First, we begin by analyzing this protocol to estimate *L*_D_ in the idealized case where the exciton creation is homogeneous (α = 0). Figure 4(a) summarizes the results, presenting the quenching efficiency curves for three regimes of diffusion: *R*_F_ = 4*r*_0_ (green), *R*_F_ = 3*r*_0_ (red), and *R*_F_ = 2*r*_0_ (blue). The data points correspond to the calculated values from the KMC results, as detailed in the Supporting File.

**Figure 4:**
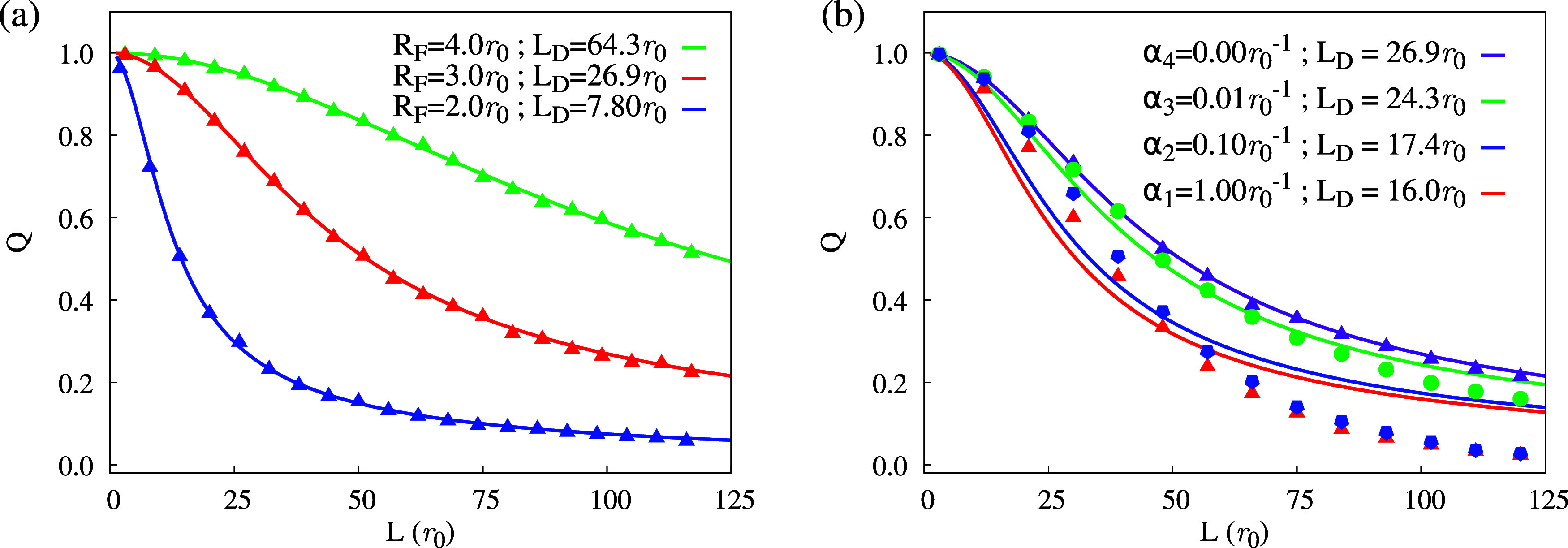
Sample of quenching efficiency curves for various diffusivities and fixed absorption coefficient (a) and varying absorption with fixed diffusivity (b). The parameter *L*_D_ was fitted within the homogeneous assumption α = 0.

All three configurations follow the same qualitative behavior: the greater the distance between the first layer and the quencher (*L*), the lower the quencher efficiency. This is expected since a greater separation for a fixed *R*_F_ reduces the chance of an exciton reaching the quencher layer. Another feature is that higher *R*_F_ is associated with a more gradual decay of *Q* when increasing *L*. This is also anticipated as the Förster radius is a measure of the local exciton diffusion. Therefore, more diffusive dynamics might allow excitons to reach the quencher layer, increasing *Q* for the same *L*.

Importantly, the lines in Figure 4(a) are the result of fitting eq 12. The overall tight agreement between data and fit is visible for all cases. Specifically, for the sampled Förster radii of 2r_0_, 3r_0_, and 4r_0_, the corresponding diffusion lengths of 8r_0_, 27r_0_, and 64r_0_ were expected according to formula [Disp-formula eq6]. This result shows that the protocol is suitable for uniform exciton generation dynamics. Therefore, in this idealized case, the quencher-based approach accurately returns the diffusion length. This is a critical check that the simulations had to satisfy since eq 12 was derived for exactly this creation condition. The clear agreement serves as a reference and corroborates the methodology.

As expected, the excellent concordance between data and the fit erodes when the generation is not homogeneous. Figure 4(b) exhibits this behavior, showing the quencher efficiency as a function of *L* for four regimes of creation with a fixed *R*_F_ = 3*r*_0_: α_4_ = 0.00*r*_0_^–1^ (purple), α_3_ = 0.01*r*_0_^–1^ (green), α_2_ = 0.1*r*_0_^–1^ (blue), and α_1_ = 1.00*r*_0_^–1^ (red). Increasing values of α are associated with an inadequate fit of eq 12. When α is high, the average exciton must travel further to reach the quencher. Effectively, this reduces *Q* compared to the homogeneous case, which in turn translates into the systematic underestimation of the fitted values of 26.9*r*_0_, 24.3*r*_0_, 17.4*r*_0_, and 16.0*r*_0_ in comparison to the theoretical *L*_D_^*F*^ = 27*r*_0_ for the fixed Förster radius 3*r*_0_. Therefore, nonhomogeneous creation consistently underestimates the measured diffusion length *L*_D_.

The primary quantity obtained experimentally in the simulated setup is the quenching efficiency as a function of the quencher position. To facilitate direct quantitative comparison, we provide the Supporting Table S1, which presents the quenching efficiencies obtained from KMC simulations across different absorption regimes and for various diffusion length values, *L*_D_^*F*^ based on Förster theory.

Although it is visible that applying the quencher-based protocol can lead to errors in the diffusion length, Figure 4(b) does not provide a numerical intuition for general cases. For a more systematic treatment, we present in Figure 5 the diffusion length percentage error heatmap, Δ*L*_D_(α; *L*_D_^*F*^), as a function of α and *L*_D_^*F*^. Regions in blue correspond to configurations in which the quencher-based prediction and actual diffusion length agree well, whereas those in yellow represent the opposite, indicating a high discrepancy. The first thing to realize is that the diffusion length range encompasses most organic compounds.^26^ Accordingly, values found in literature for experiments based on PL quenching are within the equivalent range of a few nanometers to around 100 nm.^34,41,42^ Therefore, the subsequent discussion broadly addresses the material class as a whole.

**Figure 5:**
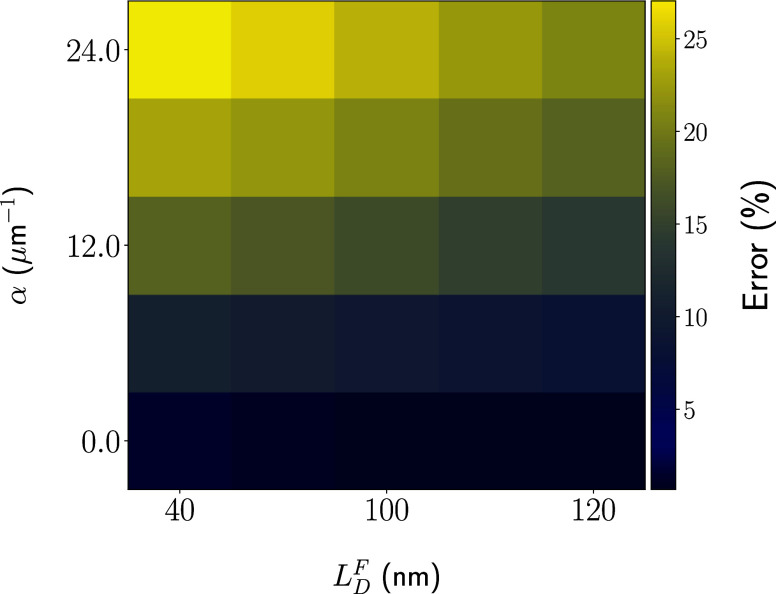
Heatmap for percentage errors Δ*L*_D_ for diverse combinations of *L*_D_^*F*^ and α.

Interestingly, the results highlight key aspects of the generation assumptions. First, for a fixed expected diffusion length (*L*_D_^*F*^), the homogeneous generation assumption leads to a monotonic increase in the percentage error. One could have anticipated this outcome since increasing α leads to progressively poorer fits in Figure 4(b). Here, the heatmap shows that this behavior is not particular to a specific diffusion regime but rather appears to be a general limitation.

However, the error magnitude is dependent on the baseline *L*_D_^*F*^. The larger the diffusion length, the lower the fitting error within the same generation scheme. The reason is that excitons with sufficiently long diffusion lengths will reach the quencher regardless of where they are generated. Consequently, in materials with high *L*_D_^*F*^, the details of the generation profile become negligible for *Q*(*L*). Therefore, the resulting errors in this setup are a relative measure governed by the interplay between α and *L*_D_^*F*^.

In general, Figure 5 indicates that errors derived from the wrong assumption of homogeneous creation can be as high as approximately 30%. This is a significant deviation, deserving attention in real implementations of quencher-based experiments. Another point is that the fits were made with an abundant and well-distributed data set, reaching an idealized experimental condition. In this way, we sought to eliminate additional error sources from the lack of data. However, realistically, the actual experiment has a limited number of points available to obtain the quencher efficiency curve.^28^ This restriction can affect the fit error and, more importantly, gives the wrong impression that the fit protocol is always suitable. Therefore, the errors reported here may be even higher when the protocol is subjected to experimental data collection restrictions.

Thus far, our treatment has been purely parametric, allowing a general discussion. However, it is important to contextualize these findings by examining real cases involving organic materials. To this end, we apply the protocol to two commonly used molecules in OPV setups: α-Sexithiophene (α-6T),^42–44^ or simply 6T, and chloroboron subphthalocyanine (BSubPcCl).^41,43–45^ The corresponding optimized geometries are illustrated in Supporting Figure S1. Extension of the analysis for these molecules requires the calculation of the FRET optoelectronic properties. The Supporting File provides further details and discusses the subsequent KMC parametrization. Moreover, a quantitative comparison between experimental *L*_D_ values and our predicted estimates for amorphous conformations is provided in the Supporting File. In short, once the molecules’ optoelectronic properties are simulated, we have access to their *R*_F_, *r*_0_ (for an amorphous substrate), and τ_emi_, which in turn allows the simulation of exciton dynamics in the quencher-bilayer setup. Notably, any system in which energy transfer is governed by FRET can be analyzed by using our methodology.

Figure 6 condenses the results for those molecules. The percentage error curves as a function of α for 6T (red) and BSubPcCl (blue) are presented in Figure 6(a). The shaded area represents the results from the parametric simulations. Both molecules lie within the area, yet they occupy opposite error extremes. Therefore, it becomes clear that the parametric results are representative of common organic materials. Markedly, based on the upper limit of the shaded area, we estimate percentage errors of up to 30% for this class of materials. Specifically, this limit applies to materials with *L*_D_^*F*^ in the range 40–60 nm and α in the range 27–30 μm^–1^.

**Figure 6:**
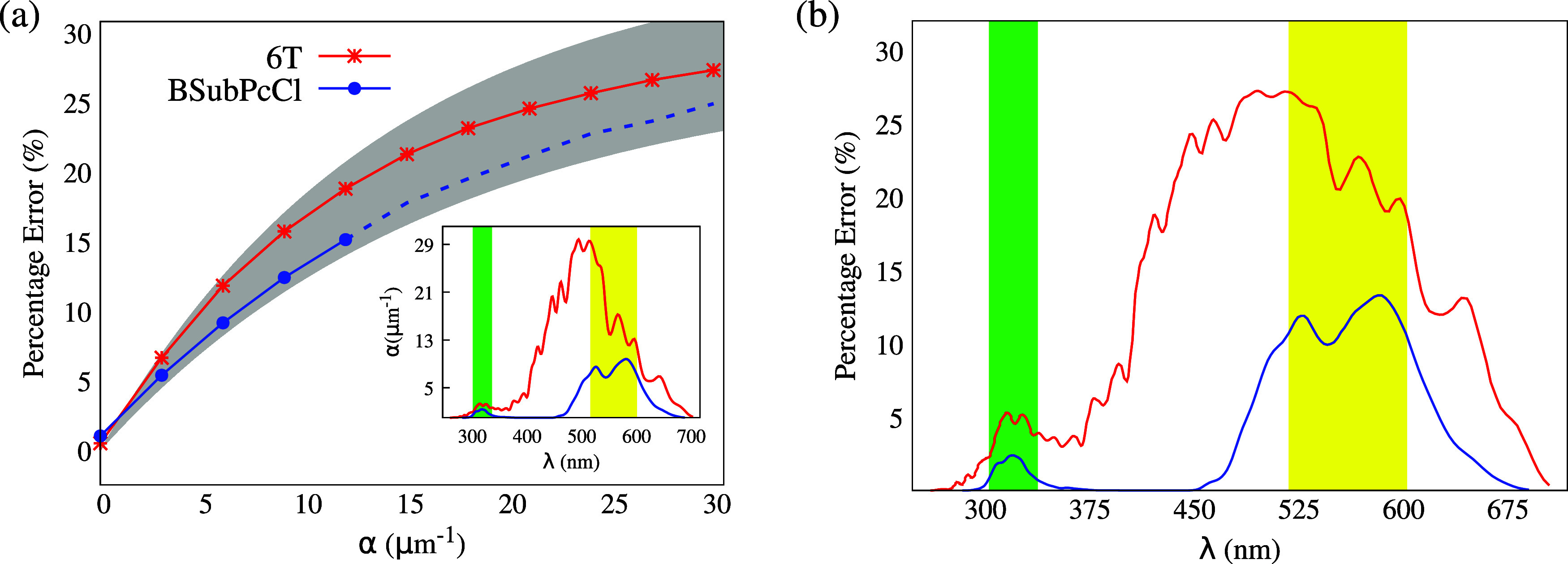
Resulting percentage errors Δ*L*_D_^*F*^ for 6T and BSubPcCl for a selected range of absorption coefficients α (a) and for a corresponding range of wavelengths in nanometers (b). The shaded region in part (a) represents the full error range of the parametric cases, while the dashed line for BSubPcCl represents the extrapolation beyond the actual absorption range. The inset in part (a) presents the dispersion relation α­(ω). Vertical shaded stripes in part (b) and in the inset of part (a) highlight simultaneous characteristic wavelength regions. The green region indicates conditions suitable for conducting the experiment with *L*_D_ errors below 5%, while the yellow region marks unsuitable conditions with errors exceeding 10%.

Our electronic structure calculations reveal that BSubPcCl has a higher *L*_D_^*F*^ than 6T. Hence, one could expect that BSubPcCl is less sensitive to the homogeneous assumption. Moreover, all of the errors increase monotonically, a behavior already identified in the parametric results. The inset in Figure 6(a) further displays (for each molecule) how α depends on the absorbed photon wavelength, λ. As seen, the α distribution is not uniform. Therefore, different incident photon energies might result in distinct absorption coefficients. This means that the diffusion length error is sensitive to the light source specifications.

To better showcase this effect, we present in Figure 6(b) the error distribution within the characteristic absorption interval of the molecules, i.e., the wavelength range of 275–700 nm. This range is relevant to the experimental setup, in which a specific wavelength laser irradiates the aggregate. Thus, the choice of the laser during preparation of the experiment is of central importance since there must be a balance between fitting accuracy and exciton generation, which comes with absorption. This fact is also illustrated in Figure 6, through the vertical shaded stripes in the inset of Figure 6(a) and in Figure 6(b). The green region highlights a wavelength range that is simultaneously suitable for conducting the experiment for both molecules. Specifically, within this region (defined by 305–340 nm), the errors in the fitted *L*_D_ are expected to remain below 5%. Conversely, conducting the experiment within the yellow region (defined by 520–605 nm) is expected to lead to *L*_D_ errors exceeding 10%.

Therefore, a guideline for selecting the excitation wavelength is established to ensure precise and consistent measurements in this setup. Prior optical characterization of the active material is a critical matter, since the choice of the excitation wavelength is based on the material’s absorptivity. As discussed, a suitable range should simultaneously produce small percentage errors and allow for the generation of excitons. Hence, the choice of the excitation wavelength is determined by the material’s absorption coefficient α­(λ), which must not be too high.

Some final remarks should be considered. Our scheme for calculating α only accounts for absorption effects. If scattering effects are significant, then the total attenuation coefficient α′ = α + α_*s*_ can only increase relative to the one considered here. Consequently, the percentage error Δ*L*_D_(α) for a fixed diffusion length is also expected to increase. Hence, our methodology predicts a lower bound for Δ*L*_D_(α; *L*_D_^*F*^) in the setup studied.

Based on the results, we conclude that the reasonable fulfillment of idealized conditions during the quencher-based *L*_D_ experiments is crucial for its precision. Therefore, to improve the *L*_D_ measurements, we identify two options: 1. reformulate the analytical *L*_D_ expression to accommodate more advanced effects in the analysis; 2. prepare the setup aiming to reach these idealized conditions as best as possible. Stating more specifically, the current protocol assumes, among other characteristics, a perfect quencher, homogeneous exciton creation, no recombination effects, and the absence of singlet–singlet annihilation. Deviation from any of these assumptions is expected to reverberate into the effective quencher count, affecting the resulting estimate for *L*_D_.

Therefore, the experiment’s reliability may be preserved by selecting materials (and instruments) that perform near these idealized conditions. Specifically, the selected quencher material must be carefully chosen to ensure a high quenching probability when paired with the material of interest. The protocol should be avoided for materials that present a strong recombination process such as ISC or singlet fission. The laser excitation regime should be chosen to prevent high singlet exciton concentrations (thus avoiding annihilation), and heterogeneous exciton generation should be minimized by selecting a pulse wavelength that matches low-probability absorbing optical transitions. Finally, the nature of the protocol is also important. Being a fit-based measurement, the nonconformity to these conditions (and the unsuitability of the predicted values) can be erroneously masked by a combination of error propagation, a lack of data points, and fit adjustment. Therefore, assessing the suitability of the method also requires extensive data collection.

A final remark concerns the parametric nature of our results, which enables a general treatment of excitonic systems. The system-specific cases of 6T and BSubPcCl were obtained under simplifying assumptions: effects such as intersystem crossing (ISC), molecular anisotropy, local symmetries, and aggregate formation were neglected. Since a full account of these mechanisms would require a dedicated study of each molecule, we instead focused on a general framework for exciton diffusion in organic materials, leaving material details aside.

## 4 Conclusion

In this work, we developed a Kinetic Monte Carlo algorithm to emulate a specific experimental setup designed to measure the exciton diffusion length in organic media. In doing so, we aimed to reproduce photoluminescence quenching curves, which in layered systems enable fitting for *L*_D_ through expression [Disp-formula eq12]. This expression was verified here to be valid only under homogeneous generation conditions, i.e., α ≈ 0.

Motivated by the relevance of the absorption coefficient α in such a setup, we were able to quantify the percentage error Δ*L*_D_^*F*^ arising from the erroneous assumption of homogeneous generation. Then, we established that the measured diffusion length is consistently underestimated in such cases. This highlights the importance of characterizing the generation scheme determined by α.

We also studied various parametric combinations of diffusivity, *L*_D_^*F*^ and α. It was revealed that the error margin associated with the homogeneous hypothesis is relative rather than absolute. Thus, this error may be negligible depending on the material itself. In this context, we considered the relevance of the laser’s wavelength λ in physical systems, such as 6T and BSubPcCl. Since our methodology only deals with absorption effects and not scattering, our results strictly predict lower bounds to Δ*L*_D_^*F*^.

In conclusion, this work provides valuable insights into diffusion length measurements. For more precise predictions, our methodology could be further extended to account for scattering effects, in addition to absorption. Nevertheless, we have demonstrated that absorption effects alone can play a significant role in layered quenching systems.

## Supplementary Material



## Data Availability

The data that support the findings of this study are available at https://zenodo.org/records/17458443.

## Notes

Theoretical details on quenching efficiency curves under the assumption of homogeneous exciton generation; a detailed description of the Kinetic Monte Carlo (KMC) algorithm used to reproduce the bilayer morphology; a table summarizing simulation parameters and outcomes; computational details for the specific molecules 6T and BSubPcCl, including electronic structure calculations; a brief explanation of the nuclear ensemble method; absorption and emission spectra; a table reporting the relevant photophysical properties; a correction to the Förster resonance energy transfer (FRET) formulas; and a statistical scheme for the approximation of intermolecular distances (PDF)
