# Supplementary material for: Influence of Nonuniform Exciton Density on Diffusion Length Measurements via Photoluminescence Quenching
Source: ACS Omega. 2025 Nov 26;10(48):58383–92. doi: 10.1021/acsomega.5c05659 (PMC12771264; doi:10.1021/acsomega.5c05659)
Supplement: Supplementary file 1 [file ao5c05659_si_001.pdf]

Supporting Information: Influence of non-uniform exciton density on diffusion length measurements via photoluminescence quenching

Bruno Guilherme Araujo Pimenta[1], Tiago de Sousa Araújo Cassiano[1], Ricardo Gargano[1], Pedro Henrique de Oliveira Neto*[1,2]

[1] *University of Brasília, Institute of Physics, 70.910-900, Brasília, Brazil*
[2] *International Center of Physics, University of Brasília, 70919-970, Brazil*

*pedrohenrique@unb.br**

Contents

1 Quencher Efficiency Curve on Homogeneous Creation **S-2**

2 KMC for Quencher-bilayer Morphology **S-2**
2.1 General Algorithm S-2
2.2 Quencher Effect S-3
2.3 Comparative Table for Measurable Quenching Efficiencies S-4

3 Electronic Structure Calculations For BSubPcCl and 6T **S-4**
3.1 Computational Details S-4
3.2 Nuclear Ensemble S-5
3.3 Short-range FRET rate corrections S-5
3.4 Photophysical Description and Materials' Details S-5
3.5 Estimate for r_0 . . . . . S-5
3.5.1 6T S-6
3.5.2 BSubPcCl S-6

1 Quencher Efficiency Curve on Homogeneous Creation

The general diffusion equation for the exciton density n on an organic photovoltaic system is:

$$\frac{\partial n(z,t)}{\partial t} = D\frac{\partial^2 n(z,t)}{\partial z^2} - \frac{n(z,t)}{\tau} + G(z,t) - S(z)n(z,t), \tag{S1}$$

which accounts for decay ($1/\tau$), generation ($G(z,t)$) and quenching rates ($S(z)$) in addition to the normal diffusion component (D). Considering the bilayer case of the quencher located at $z = L$, it is possible to approximate $S(z)$ by a Dirac delta function $\delta(L)$, which physically corresponds to an infinite quenching rate at the interface. So this term can be removed from the equation, and appropriate physical boundary conditions can be applied. Mathematically, these conditions are expressed by

$$\begin{cases} n(L) = 0 \\ \left.\dfrac{dn}{dz}\right|_{z=0} = 0, \end{cases} \tag{S2}$$

which corresponds to the absence of excitons at the interface and their confinement to the z interval $[0, L]$, with periodic boundary conditions applied in the $\hat{x}$ and $\hat{y}$ directions.

In our approach, we search for stationary solutions defined by $\frac{\partial n(z,t)}{\partial t} = 0$. This assumption is made solely to simplify the derivation, as both the time-dependent and steady-state approaches are ultimately equivalent [1]. Physically, the stationary condition is reached by adjusting the light source in a way such that the generation rate G matches the fluorescence and quenching rates. To determine this rate in a consistent way, we first solve the case for the absence of a quencher, whose differential equation takes the form

$$D\frac{\partial^2 n_0(z,t)}{\partial z^2} - \frac{n_0(z,t)}{\tau} + G = \frac{\partial n_0(z,t)}{\partial t} = 0, \tag{S3}$$

with the implied boundary conditions

$$\left\{ \left.\frac{dn_0}{dz}\right|_{z=0,L} = 0. \right. \tag{S4}$$

Since we are considering the homogeneous creation regime ($\alpha = 0$), the initial density distribution is also homogeneous and remains so in the stationary approach. Therefore, $n_0(z,t) = \rho_0$, where ρ_0 is a constant. Consequently, the second-order term in equation S3 vanishes, while G takes the value $\frac{\rho_0}{\tau}$. Thus, this is the value we shall use when returning to the conditions in equation S2. Then the solution in the presence of the quencher can be obtained using standard second-order differential equation theory which results in

$$n(z) = -\rho_0 \frac{e^{\frac{z}{L_D}} + e^{\frac{-z}{L_D}}}{e^{\frac{L}{L_D}} + e^{\frac{-L}{L_D}}} + \rho_0. \tag{S5}$$

The conclusive step involves the evaluation of simple integrals occurring in the definition of quenching efficiency used in this work, which leads directly to the result sought:

$$Q(L, L_D, \alpha = 0) = 1 - \frac{\int_0^L n(z)dz}{\int_0^L n_0(z)dz} = 1 - \frac{\rho_0 L_D(-\tanh\frac{L}{L_D} + \rho_0 L)}{\rho_0 L} = \frac{L_D \tanh\left(\dfrac{L}{L_D}\right)}{L}. \tag{S6}$$

2 KMC for Quencher-bilayer Morphology

2.1 General Algorithm

Now we present the steps taken by the algorithm to model exciton diffusion. First, a molecular lattice representing the system is defined as a three-dimensional array of positions with dimensions (N_x, N_y, N_z).

For simplicity, we consider a simple cubic lattice. In this framework, N_i and r_i denote the number of molecules and the intermolecular distance in the i-th direction, respectively, leading to a total system length of $L_i = N_i \cdot r_i$.

A fraction of the lattice is then populated with N_0 excitons, whose initial density is given by

$$\nu_0 = \frac{N_0}{V}, \tag{S7}$$

where $V = L_x L_y L_z$ represents the total volume of the system. The algorithm proceeds by randomly selecting an exciton and determining the event that it will perform. In three dimensions, there are seven possible outcomes: six transfer events (restricted to nearest neighbors) and one fluorescence event. The probability of each event occurring is given by

$$P_i = \frac{k_i}{\sum_{i=1}^{7} k_i}, \tag{S8}$$

where k_i is the Förster resonance energy transfer (FRET) rate associated with the i-th event. To establish a characteristic timescale for these events, we define the natural algorithmic time step as

$$\Delta t = \frac{1}{\sum_{i=1}^{7} k_i}. \tag{S9}$$

The simulation continues until all excitons have decayed, which, on average, occurs after a total time of $N_0 \tau_{emi} = N_0 \bar{n} \Delta t$, where $\bar{n}$ is the average number of events the exciton goes through. To obtain reliable statistical results, the entire process is repeated N_r times. Finally, the exciton diffusion length L_D is estimated by tracking the absolute displacements of excitons over the ensemble of simulations. In this case, we used $N_0 = 1$ and $N_r = 10^5$. Importantly, this approach is only possible because bimolecular events are not considered. In such regime, statistical estimates on diffusion can be obtained by considering N_r reproductions since excitons travel independently throughout the material.

2.2 Quencher Effect

In the language of the algorithm, the boundary conditions S2 implied by the the quencher layer corresponds to removing excitons that reach this layer from the simulation, which recovers $n(z = L) = 0$. We count the number N_q of excitons reaching this layer while, complementarily, registering the number N_f of excitons that go through the events of fluorescence. With these numbers, we emulate the physical quantities occuring in the experimental setup of interest, namely, the quenching efficiency $Q(L; \alpha, L_D)$ and the fluorescent efficiency $I(L; \alpha, L_D)$. The former is obtained by

$$Q(L; \alpha, L_D) = 1 - \frac{I}{I_0} = 1 - \frac{N_f(L; \alpha, L_D)}{N_0} = \frac{N_q}{N_0}, \tag{S10}$$

while the latter is given by

$$I(L; \alpha, L_D) = 1 - \frac{N_q(L; \alpha, L_D)}{N_0} = \frac{N_f}{N_0}. \tag{S11}$$

For the homogeneous case ($\alpha = 0$), equation S10 is to reproduce the fitting expression S6, while the quantities $Q(L; \alpha, L_D)$ and $I(L; \alpha, L_D)$ must satisfy the complement identity

$$I + Q = 1. \tag{S12}$$

This fact is a direct consequence of the normalized definitions S10 and S11, where the quantity $N_q + N_f$ is conserved and remains exactly equal to N_0 at all times. In this case, with the quencher present, we used $N_0 = 1000$ and $N_r = 100$.

L_D (r_0)	R_F (r_0)	α (r_0^{-1})	τ_{emi}* (ns)	L (r_0)	Q (%)
20.0	2.71	12.0	1.0	13.0	87.5
				43.0	41.0
				73.0	21.0
				103.0	12.3
60.0	3.91	6.0	1.0	13.0	98.4
				43.0	84.9
				73.0	66.7
				103.0	50.9
100.0	4.64	18.0	1.0	13.0	99.4
				43.0	93.5
				73.0	82.8
				103.0	69.6

Table S1: Quenching efficiency comparative table. $^*\tau_{\text{emi}}$ is held constant at 1.0 ns throughout the simulations. Spatial quantities are expressed in units of the lattice parameter r_0.

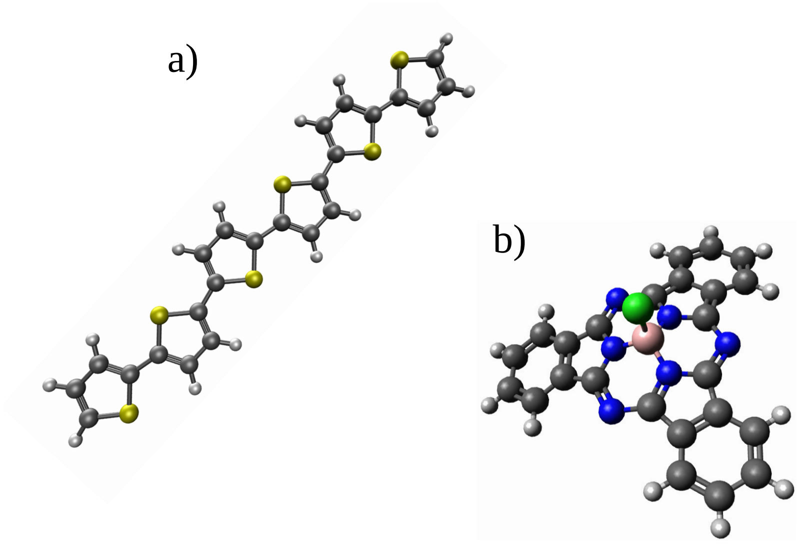

Figure S1: Optmized geometries of selected molecules: a) α-6T and b) BSubPcCl.

2.3 Comparative Table for Measurable Quenching Efficiencies

3 Electronic Structure Calculations For BSubPcCl and 6T

3.1 Computational Details

To comprehensively predict the energy and charge dynamics of the selected molecules— 6T and BSubPcCl —electronic structure calculations were conducted using Density Functional Theory (DFT) for ground-state properties and its time-dependent extension (TD-DFT) to investigate excited-state dynamics. The spectra calculations were carried out using the features built into Q-Chem [2]. Furthermore, the ensemble and ω tuning calculations were done with NEMO, and LeoX software packages [3]. Specifically, we used the functional with long-range correction ωB97X-D [4] and the basis set 6-31G(d,p).

3.2 Nuclear Ensemble

The molecular geometries within each nth electronic state are to be sampled according to the nuclear ensemble approximation [5]. Thus, the geometries in normal coordinates are sampled from the normalized distribution

$$\Omega_n = \sum_k \rho_k^T |\chi_k(q)|^2 = \prod_{j=1}^{3N-6} \left(\frac{\mu_j \omega_j}{\pi \hbar} \tanh\left(\frac{\hbar \omega_j}{2k_b T} \right) \right)^{1/2} \exp\left(- \frac{\mu_j \omega_j}{\hbar} q_j^2 \tanh\left(\frac{\hbar \omega_j}{2k_b T} \right) \right), \tag{S13}$$

where ρ_k^T is the occupancy probability for the kth vibrational level, $\chi_k(q)$ is the nuclear wavefunction in normal coordinates, the j indices refer to the normal modes with reduced mass μ_j and angular frequency ω_j, T is the temperature, $\hbar$ and k_b are the Boltzmann and Planck (reduced) constants. In the present case of a canonical ensemble, ρ_k^T takes the form

$$\rho_k^T = \frac{e^{-\frac{(E_{nk} - E_{n0})}{k_b T}}}{\sum_{k'} e^{-\frac{(E_{nk'} - E_{n0})}{k_b T}}}, \tag{S14}$$

where E_{nk} is the energy of the electronic state k in vibrational level k. For each electronic state, we've sampled 500 geometries at 300K for our spectra calculation.

3.3 Short-range FRET rate corrections

At short distances, the FRET formulae can be slightly modified to account for overlapping effects [6, 7]. In this way, the Förster rate can be written as

$$k_F = \frac{1}{\tau_{emi}} \left(\frac{R_F}{r_0 + \gamma \mu} \right)^6, \tag{S15}$$

where τ_{emi} is the exciton's radiative lifetime, r_0 is the distance between the molecules, $\gamma = 1.15e^{-1}$ is a constant expressed in terms of the electron's fundamental charge e and μ is the transition dipole moment. The corresponding diffusion length L_D^F within this modification is then expressed by [8]

$$L_D^F = r_0 \frac{R_F^3}{(r_0 + \gamma \mu)^3}. \tag{S16}$$

3.4 Photophysical Description and Materials' Details

From the spectra presented in Figure S2 and the materials' data contained in Table S2, the respective values $L_D^F = 70.4$ nm and $L_D^F = 94.3$ nm were obtained for 6T and BSubPcCl using the quantum yield correction $L_D^* = L_D \sqrt{\Phi}$ [9]. Photoluminescence quenching measurements reported in the literature yielded diffusion lengths of approximately 60 nm for 6T [10], while our calculations return 70.4 nm. On the other hand, BSubPcCl experimental estimates yield 28 nm [11] and our theoretical result equals 94.3 nm. Although our calculated result for 6T is in good agreement with experimental observations, the value obtained for BSubPcCl is significantly overestimated. This discrepancy may originate from the assumption of random orientation of dipole moments ($\kappa^2 = 2/3$), which was adopted to evaluate R_F of both molecules. While a reasonable approach when precise conformational data is not available, this approximation may not represent the exact crystalline configuration present during the experiments. For this reason, experimental comparisons must be interpreted with caution as polymorphism is prominent due to specific synthesis routes [12–18]. In the context of parametric study, the suitability of the framework (kMC with Förster Theory) is supported by its previous success on elucidating exciton diffusion phenomena for specific molecular compounds [8, 19–23].

3.5 Estimate for r_0

Our estimates for the intermolecular distancing r_0 are based on the amorphous case. With known values of molar mass (or molar volume) and macroscopic density, while approximating the molecules as spheres of radius r, the estimate is reduced to basic stoichiometry.

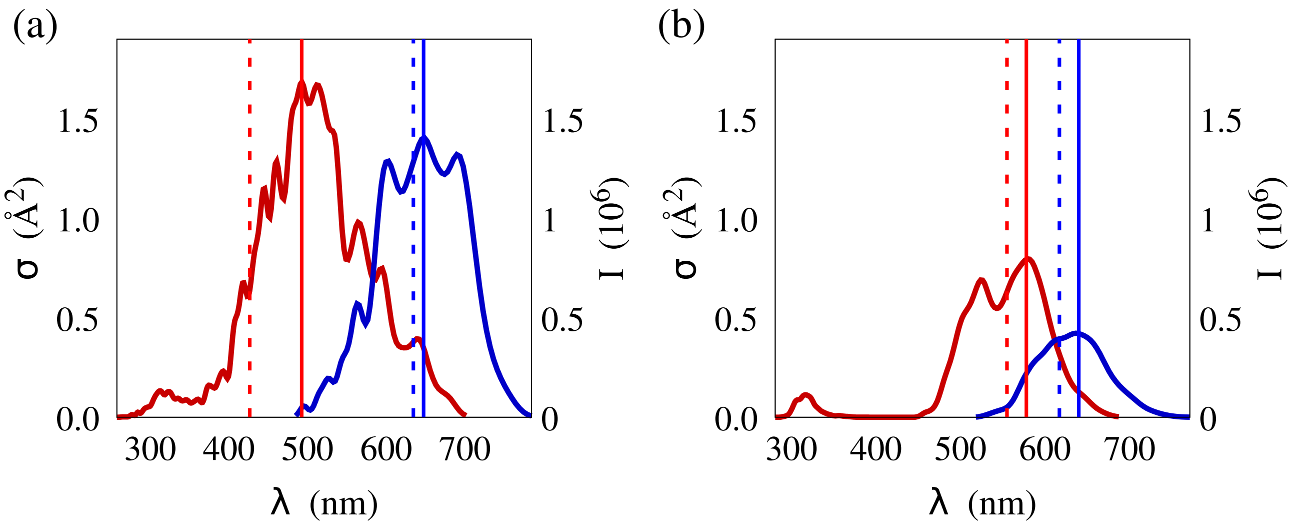

Figure S2: Absorption (red) and emission (blue) spectra for (a) α-6T and (b) BSubPcCl. Solid vertical lines of respective colors represent the calculated peaks while the dashed vertical lines represent reference experimental values.

Property	BSubPcCl		alpha-6T	
	Calculated	Experimental	Calculated	Experimental
λ_{emi} (nm)	635	622 [24]	653	640 [25]
λ_{abs} (nm)	583	560 [26]	498	432 [25]
τ_{emi} (ns)	5.1	3.3 [27]	1.0	0.7 [28]
R_F (nm)	6.3	-	6.6	-
μ (a.u.)	2.4	7.95 [29]	5.9	-
Φ	-	0.25 [27]	-	6.0 [1]
n	-	1.889 [30]	-	1.721 [31]
ρ (g/cm^3)	-	1.6 [30]	-	1.5 [32]

Table S2: Calculated and experimental data for emission peak (λ_{emi}), absorption peak (λ_{abs}), radiative lifetime (τ_{emi}), Förster radius (R_F), transition dipole moment (μ), quantum yield (Φ), refractive index (n) and density (ρ).

3.5.1 6T

6T has the molecular formula $C_{24}H_{14}S_6$, which sums to $\approx$ 494.7 g/mol [33]. Macroscopically, its density is $\approx$ 1.5 g/cm^3. Therefore, a simple stoichiometry calculation results in $3 \cdot 10^{-3}$ mol/cm^3. It follows that the average volume occupied by a 6T molecule is 560 Å^3, which for a spherical model results in an average radius $r = 5.1$ Å. Therefore, we use the intermolecular distance $r_0 = 10.2$ Å.

3.5.2 BSubPcCl

BSubPcCl has a molar volume is 265.2cm^3 [30]. It follows that the average volume occupied by a BSubPcCl molecule is 440 Å^3, which for a spherical model results in an average radius $r = 4.7$ Å. Thus, the approximated intermolecular distance is $r_0 = 9.4$ Å.

References

[1] D. E. Markov, E. Amsterdam, P. W. Blom, A. B. Sieval, J. C. Hummelen, Accurate measurement of the exciton diffusion length in a conjugated polymer using a heterostructure with a side-chain cross-linked fullerene layer, The Journal of Physical Chemistry A 109 (24) (2005) 5266–5274. doi:10.1021/jp0509663.

[2] Y. Shao, Z. Gan, E. Epifanovsky, A. T. Gilbert, M. Wormit, J. Kussmann, A. W. Lange, A. Behn, J. Deng, X. Feng, et al., Advances in molecular quantum chemistry contained in the q-chem 4 program package, Molecular Physics 113 (2) (2015) 184–215. doi:10.1080/00268976.2014.952696.

[3] L. E. de Sousa, P. de Silva, Unified framework for photophysical rate calculations in tadf molecules, Journal of Chemical Theory and Computation 17 (9) (2021) 5816–5824. doi:10.1021/acs.jctc.1c00476.

[4] Z. Chen, Y. Li, Z. He, Y. Xu, W. Yu, Theoretical investigations on charge transport properties of tetrabenzo [a, d, j, m] coronene derivatives using different density functional theory functionals (b3lyp, m06-2x, and wb97xd), Journal of Chemical Research 43 (7-8) (2019) 293–303. doi:10.1177/1747519819861626.

[5] R. Crespo-Otero, M. Barbatti, Spectrum simulation and decomposition with nuclear ensemble: formal derivation and application to benzene, furan and 2-phenylfuran, Marco Antonio Chaer Nascimento: A Festschrift from Theoretical Chemistry Accounts (2014) 89–102doi:10.1007/s00214-012-1237-4.

[6] S. R. Yost, E. Hontz, S. Yeganeh, T. V. Voorhis, Triplet vs singlet energy transfer in organic semiconductors: The tortoise and the hare, The Journal of Physical Chemistry C 116 (2012) 17369–17377. doi:10.1021/jp304389q.

[7] K. F. Wong, B. Bagchi, P. J. Rossky, Molecular excitation dynamics in condensed phases: The role of correlation in exciton transfer, The Journal of Physical Chemistry A 108 (2004) 5752–5763. doi:10.1021/jp0498174.

[8] L. E. de Sousa, F. T. Bueno, G. M. e Silva, D. A. da Silva Filho, P. H. de Oliveira Neto, Fast predictions of exciton diffusion length in organic materials, Journal of Materials Chemistry C 7 (14) (2019) 4066–4071. doi:10.1039/C9TC00153K.

[9] N. Tessler, V. Medvedev, M. Kazes, S. Kan, U. Banin, Efficient near-infrared polymer nanocrystal light-emitting diodes, Science 295 (5559) (2002) 1506–1508. doi:10.1126/science.1068155.

[10] A. Mani, J. Schoonman, A. Goossens, Photoluminescence study of sexithiophene thin films, The Journal of Physical Chemistry B 109 (11) (2005) 4829–4836. doi:10.1021/jp045916x.

[11] H. Gommans, S. Schols, A. Kadashchuk, P. Heremans, S. Meskers, Exciton diffusion length and lifetime in subphthalocyanine films, The Journal of Physical Chemistry C 113 (7) (2009) 2974–2979. doi:10.1021/jp809802q.

[12] M. Moret, A. Gavezzotti, The crystalline state of rubrene materials: intermolecular recognition, isomorphism, polymorphism, and periodic bond-chain analysis of morphologies, New Journal of Chemistry 46 (16) (2022) 7626–7637. doi:10.1039/D2NJ00861K.

[13] A. J. Cruz-Cabeza, J. Bernstein, Conformational polymorphism, Chemical Reviews 114 (4) (2014) 2170–2191. doi:10.1021/cr400249d.
URL https://doi.org/10.1021/cr400249d

[14] T. Matsukawa, M. Yoshimura, K. Sasai, M. Uchiyama, M. Yamagishi, Y. Tominari, Y. Takahashi, J. Takeya, Y. Kitaoka, Y. Mori, et al., Growth of thin rubrene single crystals from 1-propanol solvent, Journal of crystal growth 312 (2) (2010) 310–313. doi:https://doi.org/10.1016/j.jcrysgro.2009.10.048.

[15] E. Venuti, R. G. Della Valle, L. Farina, A. Brillante, M. Masino, A. Girlando, Phonons and structures of tetracene polymorphs at low temperature and high pressure, Physical Review BCondensed Matter and Materials Physics 70 (10) (2004) 104106. doi:10.1103/PhysRevB.70.104106.

[16] L. Yu, Polymorphism in molecular solids: an extraordinary system of red, orange, and yellow crystals, Accounts of chemical research 43 (9) (2010) 1257–1266. doi:10.1021/ar100040r.

[17] C. Wang, Z. Li, Molecular conformation and packing: their critical roles in the emission performance of mechanochromic fluorescence materials, Materials Chemistry Frontiers 1 (11) (2017) 2174–2194. doi:10.1039/C7QM00201G.

[18] T. Siegrist, C. Besnard, S. Haas, M. Schiltz, P. Pattison, D. Chernyshov, B. Batlogg, C. Kloc, A polymorph lost and found: The high-temperature crystal structure of pentacene, Advanced materials 19 (16) (2007) 2079–2082. doi:10.1002/adma.200602072.

[19] W. Yang, C. S. Pursglove De Castro, S. Karuthedath, Y. Firdaus, N. Alshehri, S. Chen, D. Rosas Villalva, C. E. Petoukhoff, A. Dahman, D. Baran, et al., Determining exciton diffusion length in organic semiconductors: Unifying macro-and microscopic perspectives, Advanced Energy Materials (2025) 2405322doi:10.1002/aenm.202405322.

[20] L. E. de Sousa, L. dos Santos Born, P. H. de Oliveira Neto, P. de Silva, Triplet-to-singlet exciton transfer in hyperfluorescent oled materials, Journal of Materials Chemistry C 10 (12) (2022) 4914–4922. doi:10.1039/D1TC05596H.

[21] J. Lingagouder, N. Aota, R. Nakagawa, B. Luszczynska, S. Minakata, L. E. de Sousa, P. de Silva, P. Data, Y. Takeda, Thermally activated delayed fluorescence of dibenzophenazine-cored phenazaborines in solid state: Anion modulation of photophysics, The Journal of Physical Chemistry C 128 (39) (2024) 16805–16812. doi:10.1021/acs.jpcc.4c05319.

[22] R. Saxena, T. Meier, S. Athanasopoulos, H. Bässler, A. Köhler, Kinetic monte carlo study of triplet-triplet annihilation in conjugated luminescent materials, Physical Review Applied 14 (3) (2020) 034050. doi:10.1103/PhysRevApplied.14.034050.

[23] P. Niyonkuru, A. P. Proudian, M. B. Jaskot, J. D. Zimmerman, An intermediate model for fitting triplet–triplet annihilation in phosphorescent organic light emitting diode materials, Journal of Applied Physics 132 (9) (2022) 095501. doi:10.1063/5.0102479.

[24] J. Wang, et al., Boron subphthalocyanine chloride crystalline thin film with a long range exciton diffusion length grown assisted by negative surface charges, Thin Solid Films 636 (2017) 527–531. doi:10.1016/j.tsf.2017.07.001.

[25] A. Yassar, G. Horowitz, P. Valat, V. Wintgens, M. Hmyene, F. Deloffre, P. Srivastava, P. Lang, F. Garnier, Exciton coupling effects in the absorption and photoluminescence of sexithiophene derivatives, The Journal of Physical Chemistry 99 (22) (1995) 9155–9159. doi:10.1021/j100022a031.

[26] D. Su, J. Dong, L. Wang, Z. Su, Panchromatic organic photodetectors with subpc as a non-fullerene acceptor, Materials Research Express 6 (10) (2019) 105103. doi:10.1088/2053-1591/ab3ee9.

[27] B. Del Rey, et al., Synthesis and nonlinear optical, photophysical, and electrochemical properties of subphthalocyanines, Journal of the American Chemical Society 120 (49) (1998) 12808–12817. doi:10.1021/ja980508q.

[28] R. Marks, M. Muccini, E. Lunedi, R. Michel, M. Murgia, R. Zamboni, C. Taliani, G. Horowitz, F. Garnier, M. Hopmeier, et al., Disorder influenced optical properties of α-sexithiophene single crystals and thin evaporated films, Chemical physics 227 (1-2) (1998) 49–56. doi:10.1016/S0301-0104(97)00274-7.

[29] G. Martín, et al., Subphthalocyanines and subnaphthalocyanines: nonlinear quasi-planar octupolar systems with permanent polarity, The Journal of Physical Chemistry B 106 (51) (2002) 13139–13145. doi:10.1021/jp020711e.

[30] ChemSpider, ChemSpider - CSID:10000795, accessed: 2025-03-24 22:19 (2025).
URL https://www.chemspider.com/Chemical-Structure.10000795.html

[31] Molbase, Product: [2-thiophen-2-yl-5-[5-[5-(5-thiophen-2-ylthiophen-2-yl)thiophen-2-yl]thiophen-2-yl]thiophene], accessed: 2025-03-25 (2025).
URL https://www.molbase.com/supplier/762930-product-3081816.html

[32] G. Horowitz, B. Bachet, A. Yassar, P. Lang, F. Demanze, J.-L. Fave, F. Garnier, Growth and characterization of sexithiophene single crystals, Chemistry of materials 7 (7) (1995) 1337–1341. doi:10.1021/cm00055a010.

[33] National Center for Biotechnology Information, Pubchem compound summary for cid [11340899] (2024).
URL https://pubchem.ncbi.nlm.nih.gov
